# Using Deep Neural Network Approach for Multiple-Class Assessment of Digital Mammography

**DOI:** 10.3390/healthcare10122382

**Published:** 2022-11-27

**Authors:** Shih-Yen Hsu, Chi-Yuan Wang, Yi-Kai Kao, Kuo-Ying Liu, Ming-Chia Lin, Li-Ren Yeh, Yi-Ming Wang, Chih-I Chen, Feng-Chen Kao

**Affiliations:** 1 Department of Information Engineering, I-Shou University, Kaohsiung City 84001, Taiwan; 2 Department of Medical Imaging and Radiological Science, I-Shou University, Kaohsiung City 82445, Taiwan; 3 Division of Colorectal Surgery, Department of Surgery, E-DA Hospital, Kaohsiung City 82445, Taiwan; 4 Department of Radiology, E-DA Cancer Hospital, I-Shou University, Kaohsiung City 82445, Taiwan; 5 Department of Nuclear Medicine, E-DA Hospital, I-Shou University, Kaohsiung City 82445, Taiwan; 6 Department of Anesthesiology, E-DA Cancer Hospital, I-Shou University, Kaohsiung City 82445, Taiwan; 7 Department of Medical Imaging and Radiology, Shu-Zen College of Medicine and Management, Kaohsiung City 82144, Taiwan; 8 Department of Critical Care Medicine, E-DA Hospital, I-Shou University, Kaohsiung City 82445, Taiwan; 9 Division of Colon and Rectal Surgery, Department of Surgery, E-DA Hospital, Kaohsiung City 82445, Taiwan; 10 Division of General Medicine Surgery, Department of Surgery, E-DA Hospital, Kaohsiung City 82445, Taiwan; 11 School of Medicine, College of Medicine, I-Shou University, Kaohsiung City 82445, Taiwan; 12 The School of Chinese Medicine for Post Baccalaureate, I-Shou University, Kaohsiung City 82445, Taiwan; 13 Department of Orthopedics, E-DA Hospital, Kaohsiung City 82445, Taiwan; 14 Department of Orthopedics, Dachang Hospital, Kaohsiung City 82445, Taiwan

**Keywords:** mammography, deep neural network, classification

## Abstract

According to the Health Promotion Administration in the Ministry of Health and Welfare statistics in Taiwan, over ten thousand women have breast cancer every year. Mammography is widely used to detect breast cancer. However, it is limited by the operator’s technique, the cooperation of the subjects, and the subjective interpretation by the physician. It results in inconsistent identification. Therefore, this study explores the use of a deep neural network algorithm for the classification of mammography images. In the experimental design, a retrospective study was used to collect imaging data from actual clinical cases. The mammography images were collected and classified according to the breast image reporting and data-analyzing system (BI-RADS). In terms of model building, a fully convolutional dense connection network (FC-DCN) is used for the network backbone. All the images were obtained through image preprocessing, a data augmentation method, and transfer learning technology to build a mammography image classification model. The research results show the model’s accuracy, sensitivity, and specificity were 86.37%, 100%, and 72.73%, respectively. Based on the FC-DCN model framework, it can effectively reduce the number of training parameters and successfully obtain a reasonable image classification model for mammography.

## 1. Introduction

The incidence and mortality of female breast cancer are 69.1 and 12.0 per 100,000, respectively, based on the Taiwan Health Promotion Administration in the Ministry of Health and Welfare. Among the stages of breast cancer, I and II are the most common, and the peak incidence tends to be in the younger population. Meanwhile, female cancer is headed by Breast Cancer, and its peak incidence is in forty-five-year-old to sixty-year-old female patients [1]. Furthermore, female breast cancer ranks fourth in the top ten cancer causes of death, and the mortality rate has been increasing year by year, with the mortality rate per 100,000 population increasing from 13.5% to 20.1% from 2008 to 2020. Clinically, in addition to self-examination, there are many different tools of imaging diagnosis for breast diseases, such as mammography, breast ultrasound, and breast magnetic resonance imaging [2,3,4]. In mammography, different lesions, such as breast calcifications, tumors, and cysts, can be detected using low-dose X-rays to penetrate the human body for imaging. The false-negative rate of mammography is about 10%. Small tumors may be obscured by dense tissue, resulting in the possibility that the tumor image overlaps with a large amount of normal tissue, making it difficult to distinguish. Similarly, there are also a few cases of patients with false positives. Breast ultrasound without radiation is the preferred inspection tool, especially for female cases younger than 40 years old, patients suspected of having breast lumps [5], cases with a BIRADS-0 score, or female cases of dense breasts, pregnancy, and lactation. The subject is placed in a lying position and scanned clockwise or counterclockwise with a probe at a high frequency of 7–10 MHz, which can distinguish cysts or parenchymatous tumors [6]. In the case of cysts with irregular margins or complex and recurrent ones, there is a high probability of malignancy, and further examination is required. In breast magnetic resonance imaging, a high-level examination method without radiation [7], a contrast agent containing Gadolinium (Gd) is injected into the patient’s intravenous veins to facilitate the observation of the distribution of breast blood vessels on the image.

Based on a literature review, there is feasible and challenging technology for computer-vision-assisted detection and analysis of breast cancer. In traditional technology, the study published by Arden Sagiterry Setiawan et al. in 2015 proposed to use LTEM (Law’s Texture Energy Measure) as the method of texture feature extraction [8]. The study adopted an Artificial Neural Network (ANN) using two-layer feedforward backpropagation for breast imaging classification. The results of the study show that LAWS provides better accuracy compared to other similar methods (such as GLCM). LAWS provides 83.30% accuracy for benign or malignant images classification. The GLCM has only 72.20% accuracy for normal/abnormal classification and 53.06% for benign/malignant classification. In the 2017 study by Yuchen Qiu et al. [9], the study report shows total 560 regions of interest (ROI) mammography images that were extracted, the input image is 512 × 512 pixels, and the 64 × 64 pixels of ROI are taken out as the targets of image feature extraction. It is followed by the 8 layers of the designed deep learning network layer, including 3 max pooling layers for automatic feature extraction and a multilayer perceptron (MLP) as a classifier to process ROI. The results showed that the AUC of the model obtained was 0.790 ± 0.019. A computer-aided diagnosis (CAD) system is an affordable, readily available, fast, and reliable source of early diagnosis [10,11].

Mammography is one of the most commonly used clinical methods for screening breast-related diseases. In studies, there are many applications in the domain of artificial intelligence. Meanwhile, different architectures of convolutional networks have also been developed to interpret different types of images [12,13] such as those obtained by computed tomography, magnetic resonance imaging, and ultrasound. In recent years, the methods of image recognition by artificial intelligence have been actively developed with higher sensitivity and specificity than CAD [14,15,16,17]. This not only enables radiologists to reduce errors in diagnosis results but also reduces the time for interpretation. In 2019, Alejandro Rodriguez-Ruiz et al. used deep learning convolution neural networks to classify and detect calcification and soft tissue lesions [18]. In 2020, Thomas Schaffter et al. developed a deep learning model called Vanilla U-Net, using U-Net as a base model, for tiny segment lesions in mammography images [19]. In 2021, José Luis Raya-Povedano et al. used artificial intelligence in digital mammography (DM) and digital breast tomosynthesis (DBT), using the system to detect suspicious lesions and mark them on each image [20]. 

This study uses the clinically collected mammograms and the BIRADS grading report as image datasets to establish the classification model with artificial intelligence technology. The main aim is to adopt the fully convolutional dense connection network (FC-DCN) to create the classification model. Then, the model performance can be evaluated in the small-sample-size mammography images. It can provide appropriate classification results to assist clinicians and reduce the time required for clinical interpretations.

## 2. Methodology

### 2.1. Mammography Image Collection and Description

**Figure 1:**
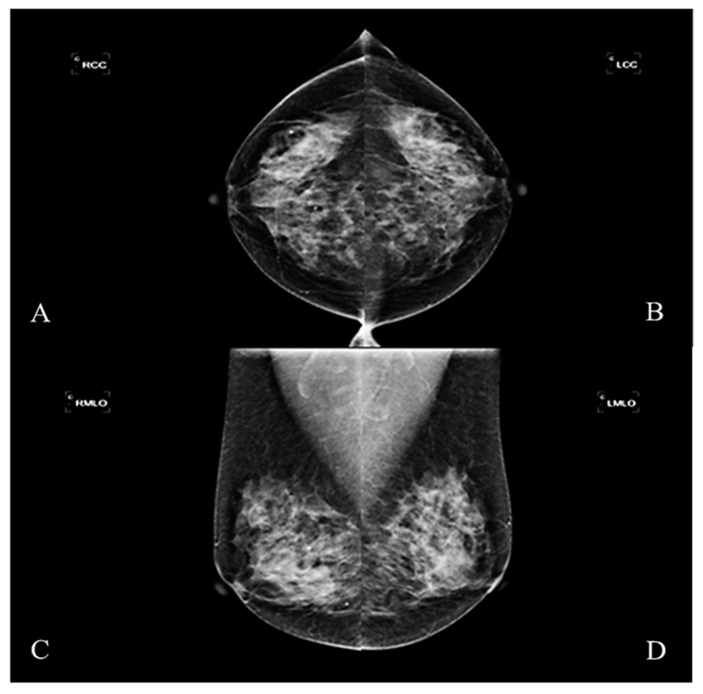
The mammographic image. (**A**) Right craniocaudal view (RCC), (**B**) left craniocaudal view (LCC), (**C**) right mediolateral oblique view (RMLO), (**D**) left mediolateral oblique view (LMLO).

This study was designed as a retrospective group experiment and collected mammography cases and diagnostic reports from 2016–2017, which had been reviewed by the Medical Ethics Committee of E-DA Hospital (EMRP-107-031). The imaging instrument used was Hologic Lorad Selenia. Each case in the study was irradiated with four images from multiple angles. These were the right craniocaudal view (RCC), the left craniocaudal view (LCC), the right mediolateral oblique view (RMLO), and the left mediolateral oblique view (LMLO) (Figure 1). Most breast tissue can be sighted in a single image with MLO View, such as pectoralis major and axillary lymph nodes. The CC view can avoid medial tissue missed with the MLO view.

In clinical practice, the American College of Radiology (ACR) established the Breast Image Reporting and Data Analyzing System (BI-RADS), in order to ensure the consistency of inspection reports and facilitate the collection and comparison of inspection results. The grading of results is based on the image characteristics of the tumor, including shape, appearance, density, edge, calcification pattern, distribution, and the symmetry of the breast tissue on both sides determined by mammography. The cases of BIRADS-0 were excluded from the conditions for acceptance of this study. Cases of incomplete examinations and additional images required for interpretation were also excluded. This study takes cases of BIRADS-4 and 5 as the subjects. Most cases of BIRADS-6, diagnosed with breast cancer, had surgery on or under treatment. They were also excluded. The tissue of the subjects being operated on might be destroyed and affect feature extraction and texture analysis. 

In the study, different pieces of images of mammography on the left and right are extracted, which excluded local surgical resection and obvious skin folds. The study included the images from 150 cases based on the pathology report diagnosed by the radiologist, including 50 negative cases (BIRADS-1) and 100 positive cases (BIRADS-4 and BIRADS-5), and the data were expanded to 5400 images through data augmentation technology [16,21]. Each image was augmented by rotating, horizontally flipping, and vertically flipping. DICOM was the file format of the mammography image, and the pixel size was resized to 1024 × 1024. All the images are split into 7:3 and 5:5 to be the training set and the testing set, respectively. This split percentage was considered based on a review of the relevant literature [22,23,24,25]. In the positive cases, the lesions included calcifications, well-defined or localized masses, radially shaped masses, other and uncertain defined masses, structural distortions, and asymmetry tissue. 

### 2.2. Experimental Design

**Figure 2:**
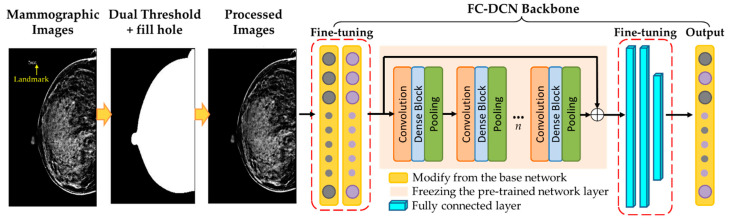
The classification architecture for mammography image.

A fully convolutional densely connected network (FC-DCN) model was developed for mammography image classification. The experimental design included image preprocessing, pre-trained model, transfer learning, model weighted calculation, etc. The research process is shown in Figure 2, and the practical steps are as follows.

Step 1. Collecting mammography images.
Step 2. Removing the imaging marker (such as right/left side notation) and embedding the image-processing technique.
Step 3. Dividing collected images of breasts into training group and test group randomly.
Step 4. Training the classified model by FC-DCN with transfer learning technology and fine-tuning the model weight.
Step 5. Testing different parameters by FC-DCN model to find the best parameters for mammography image interpretation.
Step 6. Using the accuracy, sensitivity, and specificity of the testing set as the indicators of validation performance.

After importing the mammography images, the background markers of patient data and location labels were removed. The unnecessary factors that might interfere with the model analysis in the region of breast image must be removed, including the radiologist’s code, the markers on the left and right, the name of hospital, and dose values used. To remove the background marker, the double-threshold method was used [26,27]. The image intensity values less than five and higher than 4090 were removed. Most of the subjects with values above 4090 were artificial markers. After thresholding, there were small holes in the screen. Therefore, the hollow space in the image was filled with the image fill technology [28]. Then, the filled-in image was the region of interest to specify the area in the image and achieve removal of background marker. Next, the model training was completed with the preprocess images input into the architecture of the FC-DCN.

### 2.3. Deep Transfer Learning Model

In 2017, Huang et al. published a research report at the IEEE Conference on Computer Vision and Pattern Recognition (CVPR) [29]; the method they published mainly solves the issues of the vanishing gradient problem, feature propagation, feature recyclability, and reducing the number of parameters. Hence, it can be used on personal computer or NB platforms. The authors named the architecture of their network as a dense convolutional network on account of its densely connected nature. All information input is allowed to be used among layers, and the information in any layer could be connected to subsequent layers.

This study’s pre-trained model was used as the network backbone and was also built with the transfer learning technique. There are three emphases in transfer learning: first, modifying the input layer; second, retraining the weights; third, modifying the output layer. It included image input layer, convolution layer, pooling layer, dense block and transition layer, and classification layer. Each layer’s design structure is introduced below. The training parameter is listed in Table 1.

In the input layer of the image, the image pixel size is adjusted from 1024 × 1024 to 224 × 224 pixels, and the image is input to the next layer, which is the first convolutional layer, for which convolution kernel size is 7 × 7, the stride is 2, and the output image size is 112 × 112. The eigenvalues of each region in the image are collected by the convolution layer, and the weights of each point in the convolution kernel are multiplied based on the specified convolution kernel size and stride to obtain the feature map. The pooling layer, for which the kernel size is 3 × 3, the stride is 2, and the size of output image is 56 × 56, is connected after the convolution layer (in this study, we used max pooling). Pooling is another important concept in convolutional networks and is actually a form of downsampling, which reduces the dimensionality of each feature map and retains eigen features by reducing the size of the input image—most often by half—and avoids redundant calculations and speeds up the efficiency of system operation by decreasing the parameters required for subsequent layers. It does not affect the results, with minor differences in the adjacent areas of pixels in the image to improve the consistency of the output and reduce the situation of overfitting. 

In FC-DCN, it is most important to design many dense blocks in the architecture to improve the compactness of the model further and reduce the number of feature maps in the transition layer. The dense block contains the number of m characteristic mapping. It lets the lower transition layer generate the number of [*θ*m] output feature maps, where 0 < *θ* ≤ 1, which is called the compressibility factor. As *θ* = 1, the number of feature maps remains unchanged, and it crosses the transition layer. In this study, the network structure with four dense blocks is used. A batch normalization layer (BN Layer), a rectified linear unit layer (ReLU Layer), and a convolutional layer with a kernel size of 3 × 3 are contained in dense blocks. For convolutional layers with a kernel size of 3 × 3, each side of the input is zero stuffing in order to keep the feature map size fixed. The final output layer includes the global average pooling layer (GAP Layer), fully connected layer, and normalization layer. As mentioned, adopting the fully connected layer to integrate the results of the convolution and pooling operations makes it possible to extract features. It can reduce image parameters separately and input the feature information into the fully connected layer for classification. Each connection has its own independent and different weight value. The softmax function transforms the vector z into another K-dimensional vector σ(z) to make each element in the range of (0, 1), and the sum of all elements is 1 (Equation (1)). The softmax function is usually placed in the last layer of the neural network.

(1) $$\sigma \left(z_{j}\right)=\frac{e^{z_{j}}}{\sum_{k=1}^{K}e^{z_{k}}}\;\;\;\;for\;j=1,\ldots ,k$$

The output value of the parameter value in a neural node is represented by “*z*_*j*_” after performing the weight calculation of class j, and σ(z)j is the probability that the sample vector Z belongs to the *j*th class; that is to say, the input of the function is obtained from different linear functions of K as a result, when the input is Z, the probability that the predicted class is j is σ, and the sum of the probabilities of all predicted classes is 1. The softmax function is usually placed in the last layer of the neural network. The outputs of all nodes in the last layer are passed through the exponential function, the results are added as the denominator, and the individual outputs are used as the numerator. The cross-entropy loss of the multi-class classification problems with mutually exclusive classes is calculated with the classification layer, which is also the last output layer in the network layer. In addition, the output of the function value is based on the softmax layer as the classification basis to classify it to the correct category. Meanwhile, the cross-entropy function is used to assign each input to one of the K mutually exclusive classes for the output result of 1-K encoding scheme. This function uses its backpropagation to correct the weights and biases in the hidden layer. This correction and optimization improve the accuracy of neural network classification. In Equation (2), “N” is the number of samples, “K” is the number of species, t_ij_ is the indication that the i^th^ sample belongs to the j^th^ species, and y_ij_ is the output of sample i of species j, which in this case is the value of the softmax function. That is, it is the probability that the i^th^ input to the network is associated with category j.

(2) $$\mathrm{loss}=-\sum_{i=1}^{N}\sum_{j=1}^{K}t_{ij}ln(y_{ij})$$

### 2.4. Evaluation Criteria

The image samples are randomly obtained for each iteration to build a model, with 7:3 and 5:5 as training and testing sets. Furthermore, the sensitivity and specificity of the test set are used as validation performance indicators.

(1) Sensitivity: it is defined as TP/(TP + FN). It represents the proportion of positive samples in the test set when the model predicts that the samples are positive in the true judgment. It is also known as true-positive rate (TPR); the opposite is false-positive rate (FPR), which is defined as FP/(FP + TN).
(2) Specificity: it is defined as TN/(TN + FP), which represents the proportion of the true-negative samples in the test set when the model predicts that the samples are negative; it is also known as true-negative rate (TNR); its opposite is false-negative rate (FNR), which is defined as FN/(TP + FN).
(3) Accuracy: it is defined as (TP + TN)/(TP + FN + FP + TN), which represents the proportion of true positives and true negatives in all samples when the model predicts that the samples in the test set are true positives and true negatives.

The sensitivity and specificity are used to measure the effectiveness of the classifier. In above, TP means true-positive fraction, TN means true-negative fraction, FN means false-negative fraction, and FP means false-positive fraction. For the mentioned criteria, higher values indicate better classification performance.

## 3. Results

In this study, FC-DCN was used to classify the positive and negative lesions based on BIRADS grades of breast images. Meanwhile, 5:5 and 7:3 of the data were used as the training and testing sets, respectively. In each iteration, image samples were randomly obtained to build a model, and then the test set was used to verify the model. Sensitivity, specificity, and accuracy were used as the indicators of the validation performance.

The interaction between batch size and the number of iterations is discussed separately, when the training and testing dataset is set to 7:3. According to the experimental data, the batch sizes of 20, 30, 40, 50, 60, 70, and 100 and iteration times of 100, 50, 100, 50, 50, 50, and 100 have better results, and their accuracies are 0.818, 0.864, 0.727, 0.727, 0.682, 0.773, and 0.727, respectively (Table 2). The results show that increasing the number of iterations does not actually improve the performance of the model, for the model reached the condition (state) of convergence. In addition, in the case where the two-fold cross validation method is used and the training and testing dataset is set to 5:5, the connection between the batch size and the number of iterations was ambiguous. According to the data in the experiment, the accuracies are 0.658, 0.632, 0.684, 0.684, 0.684, 0.684, and 0.789 for batch sizes of 20, 30, 40, 50, 60, 70, and 100 with the iteration times of 60, 60, 60, 50, 60, 50, and 50, respectively (Table 3). The result does not show a higher batch size that can obtain a better result.

**Figure 3:**
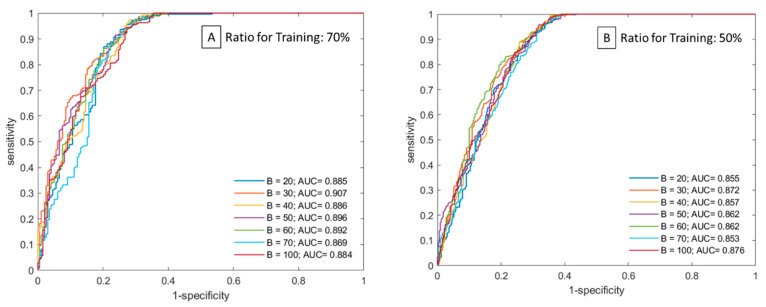
Receiver operating characteristic curves of the classified model on each batch size. Picture (**A**) presents by split ratio of 7:3. Picture (**B**) presents by split ratio of 5:5.

The performance of this multi-class classification issue can be illustrated utilizing the receiver operating characteristics (ROC) curve. The performance of a classification model at all classification thresholds can be shown on a graph called a ROC curve. This curve outlines two different parameters such as true-positive rate and false-positive rate. The term true-positive rate is used to represent sensitivity. The term false-positive rate is used to represent 1-specificity. The ROC curve is plotted with 1-specificity on the y-axis against the sensitivity on the x-axis. In Figure 3, ROC curves are plotted for every model. The best AUC achieved by the FC-DCN model at a ratio equal to 70% is 0.907, and the best AUC at a ratio equal to 50% is 0.876. This shows our model has excellent discrimination.

**Figure 4:**
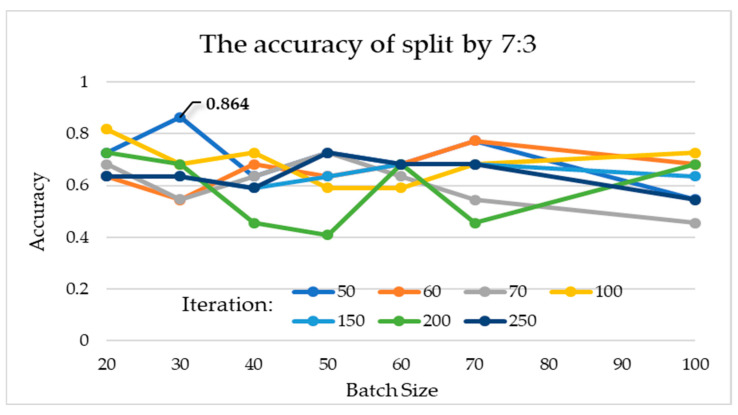
Experimental results for different batch sizes and iterations at a split ratio of 7:3.

**Figure 5:**
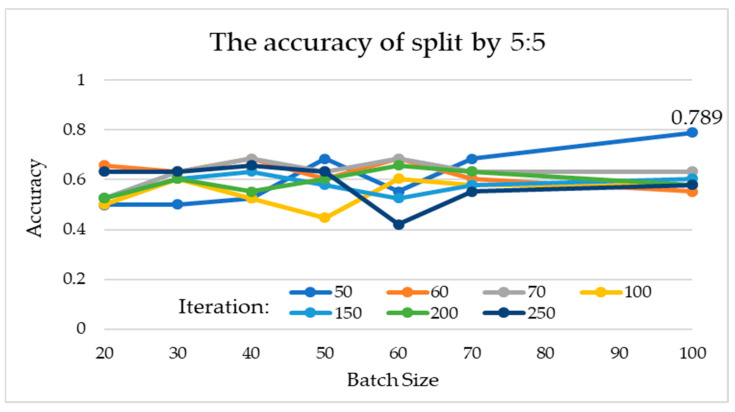
Experimental results for different batch sizes and iterations at a split ratio of 5:5.

According to the statistical indicators, different batch sizes and iterations were used for training and testing in 7:3 and 5:5 of the data. The experiments were conducted to compare the interactions among 7 batch sizes and 7 iteration parameter combinations. In 7:3 of the data, the best parameter-construction model is to use batch size 30 and iterations 50, for which sensitivity is 100%, specificity is 72.73%, and accuracy is 86.37%. In 5:5 of the data, the best parameter construction model is to use a batch size 100 and iterations 50, for which sensitivity is 78.95%, the specificity is 78.95%, and the accuracy is 78.95%. Figure 4 and Figure 5 show each accuracy of batch size from 20~100. The experimental results show that it does not improve sensitivity, specificity, or accuracy to increase the number of iterations or batch size. However, it will be related to the split ratio.

## 4. Discussion

Based on the above experiments, 7:3 and 5:5 of each group were divided into the training set and the testing set. In each iteration, image samples were randomly obtained to build a model, and the confusion matrix was used to calculate the sensitivity and specificity of the test set as the indicator of the verification performance. When the training set is 7:3 to use mini-batch size 30 and iterations 50, the sensitivity is 100%, the specificity is 72.73%, and the accuracy is 86.37%.

This study found that in the model training of mammography data, even increasing the number of iterations does not actually increase the accuracy, so there is no existing overfitting problem. In the experimental results, the batch size is relatively important. Increasing the batch size can more effectively provide more information for the model, so that better results can be obtained.

This study confirms that FC-DCN can successfully achieve good performance with a small number of parameters and a small amount of computation; moreover, it is an effective method to use FC-DCN to identify specific breast images. FC-DCN introduces direct connections between any two layers with the same characteristic mapping size. It can be found that FC-DCN does not show optimization difficulties when it is extended to hundreds of layers. In the relevant literature (Table 4), the model established in this study can be used for small-sample-size data to obtain over 85% classification accuracy. In addition, regarding the ratio of data split, this research method can still obtain nearly 80% accuracy under the condition that the data split ratio is 5:5.

Table 4 compares the same model architecture with Medeiros et al. The authors used the ROI selection method for dataset preprocessing. ROI extraction is an accepted technique. This study still considers using the entire image for model training. The whole image can provide more feature information. Hence, the model can have more data to learn during training. Therefore, it can obtain better performance. However, the disadvantage is the need to spend more training time. Furthermore, the sample size is also one of the reasons to use the whole image. Based on simple connection rules, FC-DCN integrates the properties of feature maps and learns deeply with diverse depths. In addition, it allows features to be reused throughout the network; hence, learning can be more compact. FC-DCN is a good feature extractor due to its compact internal representation and reduced feature redundancy. For this reason, CNN models developed by artificial intelligence can be widely used clinically in mammography images, even for different image tools.

In this paper, the deep learning algorithm designed belongs to “weak AI” in the field of artificial intelligence. In weak AI, human intervention is relied on to define the parameters of learning algorithms and provide relevant training data to ensure accuracy. In recent years, scientists have worked hard to move towards “strong AI”. In the future, this research can introduce semi-automatic analysis methods. First, the model will be used for the preliminary classification, and then experts will conduct secondary screening. In addition to reducing the degree of human intervention, it will gradually lead to stronger AI development.

## 5. Conclusions

This study proposes a deep learning FC-DNN method for stage classification from mammography images. Since deep learning methods do not require manual feature processing, the model performs exceptionally well compared to traditional image-processing techniques. The excellent performance of mammography detection is supported by accurate classification results. As a result, this technology can help during image interpretation screening, reducing the error rate and decreasing the computational time. Meanwhile, this study can be used to provide a theoretical framework for an assisted diagnosis system.

The deep neural network can be categorized into supervised learning, unsupervised learning, and semi-supervised learning. The supervised learning task is mostly accomplished by classifying predefined labeled training data (also known as “ground truth”). On the other hand, unsupervised learning is quite automated, as the network can automatically learn the correct answers from a huge amount of data, without requiring predefined labels [34]. Semi-supervised learning is the combination of two approaches by relatively smaller amounts of unlabeled data. In this study, supervised machine learning approach is used.

According to this research, using a pre-trained model could reduce the time spent on the new CNN model development. However, to use the pre-trained model, it needs to conform to the model architecture, and the model weight must be fine-tuned. The fine-tuning needs to be carried out before training. In fine-tuning, the image size of source data and the number of output categories need to be corrected.

In this paper, the dense block is selected as the model base architecture. The reason is that dense block can effectively alleviate the problem of model gradient vanishing [35,36,37], making backpropagation easier and the model convergence effect better. FC-DCN retains important features more comprehensively from the initial layer to the final output through feature reuse. Finally, fewer model training parameters exist because the old feature maps do not need to be relearned.

Based on the above, the method developed in this study can be applied to data types with a small number of samples. The established model can provide clinically useful reference information, speed up clinical operations, and reduce human misjudgment. In addition, the research also tested the stability of the model architecture through different data split ratios and found that even in 50% of the cases, an accuracy of nearly 80% can be obtained. In the future, we will continue to develop lightweight models to increase the possibility of clinical application.

## Figures and Tables

**Table 1:** The investigated training model.

Item	Content
Ratio for training	70%, 50%
Batch Size	20, 30, 40, 50, 60, 70, 100
Epoch Size	50, 60, 70, 100, 150, 200, 250
Learning rate	0.0001
Optimizer	sgdm

**Table 2:** The experiment results in investigated batch size by split percentage 7:3.

Batch Size	Accuracy	Sensitivity	Specificity	Kappa	AUC
20	0.818	0.909	0.727	0.634	0.885
30	0.864	1.000	0.727	0.657	0.907
40	0.727	0.546	0.909	0.631	0.886
50	0.727	0.909	0.546	0.654	0.896
60	0.682	0.727	0.636	0.625	0.892
70	0.773	0.818	0.727	0.619	0.869
100	0.727	0.818	0.636	0.627	0.884

**Table 3:** The experiment results in investigated batch size by split percentage 5:5.

Batch Size	Accuracy	Sensitivity	Specificity	Kappa	AUC
20	0.658	0.526	0.790	0.631	0.855
30	0.632	0.790	0.474	0.613	0.872
40	0.684	0.737	0.632	0.606	0.857
50	0.684	0.895	0.474	0.576	0.862
60	0.684	0.737	0.632	0.630	0.862
70	0.684	0.579	0.790	0.621	0.853
100	0.789	0.789	0.789	0.615	0.876

**Table 4:** Comparison with other works for mammography image classification.

Reference	Categories	Cases/Extend	Split Scale	Model	Accuracy
Medeiros et al. [30]	2	10239/no	8:2	DenseNet201-MLP	0.634
Lenin et al. [31]	6	18000/no	8:2	nasnet-m-TL	0.812
Yang et al. [32]	2	384/1173	8:2	MommiNet-v2	0.910(AUC)
Boumaraf et al. [33]	4	500/2620	6:4	--	0.845
Our	3	150/5400	7:3	FC-DCN	0.864
			5:5		0.789

## Data Availability

Not applicable.

## References

[1] Ho M.L., Hsiao Y.H., Su S.Y., Chou M.C., Liaw Y.P. (2015). Mortality of breast cancer in Taiwan, 1971–2010: Temporal changes and an age-period-cohort analysis. J. Obstet. Gynaecol..

[2] Khodjaeva D.I. (2021). Magnetic-resonance imaging in the diagnosis of breast cancer and its metastasis to the spinal column. Sci. Prog..

[3] Murtaza G., Shuib L., Abdul Wahab A.W., Mujtaba G., Nweke H.F., Al-garadi M.A., Zulfiqar F., Raza G., Azmi N.A. (2020). Deep learning-based breast cancer classification through medical imaging modalities: State of the art and research challenges. Artif. Intell. Rev..

[4] Beutel J., Kundel H.L., Kim Y., Van Metter R.L., Horii S.C. (2000). Handbook of medical imaging.

[5] Sehgal C.M., Weinstein S.P., Arger P.H., Conant E.F. (2006). A review of breast ultrasound. J. Mammary Gland. Biol. Neoplasia.

[6] Brem R.F., Lenihan M.J., Lieberman J., Torrente J. (2015). Screening breast ultrasound: Past, present, and future. Am. J. Roentgenol..

[7] Gallagher F.A., Woitek R., McLean M.A., Gill A.B., Manzano Garcia R., Provenzano E., Riemer F., Kaggie J., Chhabra A., Ursprung S. (2020). Imaging breast cancer using hyperpolarized carbon-13 MRI. Proc. Natl. Acad. Sci. USA.

[8] Setiawan A.S., Wesley J., Purnama Y. (2015). Mammogram classification using law’s texture energy measure and neural networks. Procedia Comput. Sci..

[9] Qiu Y., Yan S., Gundreddy R.R., Wang Y., Cheng S., Liu H., Zheng B. (2017). A new approach to develop computer-aided diagnosis scheme of breast mass classification using deep learning technology. J. X-ray Sci. Technol..

[10] Doi K. (2007). Computer-aided diagnosis in medical imaging: Historical review, current status and future potential. Comput. Med. Imaging Graph..

[11] Sadaf A., Crystal P., Scaranelo A., Helbich T. (2011). Performance of computer-aided detection applied to full-field digital mammography in detection of breast cancers. Eur. J. Radiol..

[12] Kale M.C., Fleig J.D., İmal N. (2013). Assessment of feasibility to use computer aided texture analysis based tool for parametric images of suspicious lesions in DCE-MR mammography. Comput. Math. Methods Med..

[13] Norman B., Pedoia V., Noworolski A., Link T.M., Majumdar S. (2019). Applying densely connected convolutional neural networks for staging osteoarthritis severity from plain radiographs. J. Digit. Imaging.

[14] Huang S., Yang J., Fong S., Zhao Q. (2021). Artificial intelligence in the diagnosis of COVID-19: Challenges and perspectives. Int. J. Biol. Sci..

[15] Balasubramaniam V. (2021). Artificial intelligence algorithm with SVM classification using dermascopic images for melanoma diagnosis. J. Artif. Intell. Capsul. Netw..

[16] Chlap P., Min H., Vandenberg N., Dowling J., Holloway L., Haworth A. (2021). A review of medical image data augmentation techniques for deep learning applications. J. Med. Imaging Radiat. Oncol..

[17] Kontos D., Conant E.F. (2019). Can AI help make screening mammography “lean”?. Radiology.

[18] Rodriguez-Ruiz A., Lång K., Gubern-Merida A., Broeders M., Gennaro G., Clauser P., Helbich T.H., Chevalier M., Tan T., Mertelmeier T. (2019). Stand-alone artificial intelligence for breast cancer detection in mammography: Comparison with 101 radiologists. JNCI J. Natl. Cancer Inst..

[19] Schaffter T., Buist D.S., Lee C.I., Nikulin Y., Ribli D., Guan Y., Lotter W., Jie Z., Du H., Wang S. (2020). Evaluation of combined artificial intelligence and radiologist assessment to interpret screening mammograms. JAMA Netw. Open.

[20] Raya-Povedano J.L., Romero-Martín S., Elías-Cabot E., Gubern-Mérida A., Rodríguez-Ruiz A., Álvarez-Benito M. (2021). AI-based strategies to reduce workload in breast cancer screening with mammography and tomosynthesis: A retrospective evaluation. Radiology.

[21] Zhao T., Liu Y., Neves L., Woodford O., Jiang M., Shah N. Data augmentation for graph neural networks. Proceedings of the AAAI Conference on Artificial Intelligence.

[22] Boudouh S.S., Bouakkaz M. Breast Cancer: Using Deep Transfer Learning Techniques AlexNet Convolutional Neural Network For Breast Tumor Detection in Mammography Images. Proceedings of the 2022 7th International Conference on Image and Signal Processing and their Applications (ISPA).

[23] Rehman K.U., Li J., Pei Y., Yasin A., Ali S., Saeed Y. (2021). Architectural Distortion-Based Digital Mammograms Classification Using Depth Wise Convolutional Neural Network. Biology.

[24] Wang Y., Qi Y., Xu C., Lou M., Ma Y. (2022). Learning multi-frequency features in convolutional network for mammography classification. Med. Biol. Eng. Comput..

[25] Rehman K.U., Li J., Pei Y., Yasin A., Ali S., Mahmood T. (2021). Computer vision-based microcalcification detection in digital mammograms using fully connected depthwise separable convolutional neural network. Sensors.

[26] Hou W., Zhang D., Wei Y., Guo J., Zhang X. (2020). Review on computer aided weld defect detection from radiography images. Appl. Sci..

[27] Zhang J., Guo Z., Jiao T., Wang M. (2018). Defect detection of aluminum alloy wheels in radiography images using adaptive threshold and morphological reconstruction. Appl. Sci..

[28] Soille P. (1999). Morphological Image Analysis: Principles and Applications.

[29] Hu J., Shen L., Sun G. Squeeze-and-excitation networks. Proceedings of the IEEE Conference on Computer Vision and Pattern Recognition (CVPR).

[30] Medeiros A., Ohata E.F., Silva F.H., Rego P.A., Reboucas Filho P.P. An approach to BI-RADS uncertainty levels classification via deep learning with transfer learning technique. Proceedings of the 2020 IEEE 33rd International Symposium on Computer-Based Medical Systems (CBMS).

[31] Falconí L., Pérez M., Aguilar W., Conci A. Transfer learning and fine tuning in mammogram bi-rads classification. Proceedings of the 2020 IEEE 33rd International Symposium on Computer-Based Medical Systems (CBMS).

[32] Yang Z., Cao Z., Zhang Y., Tang Z., Lin X., Ouyang R., Wu M., Han M., Xiao J., Huang L. (2021). MommiNet-v2: Mammographic multi-view mass identification networks. Med. Image Anal..

[33] Boumaraf S., Liu X., Ferkous C., Ma X. (2020). A new computer-aided diagnosis system with modified genetic feature selection for bi-RADS classification of breast masses in mammograms. BioMed Res. Int..

[34] Suen H.-Y., Hung K.-E., Lin C.-L. (2019). TensorFlow-based automatic personality recognition used in asynchronous video interviews. IEEE Access.

[35] Priya K., Peter J.D. (2022). A federated approach for detecting the chest diseases using DenseNet for multi-label classification. Complex Intell. Syst..

[36] Rathore Y.K., Janghel R.R. (2022). Prediction of Stage of Alzheimer’s Disease DenseNet Deep Learning Model. Next Generation Healthcare Systems Using Soft Computing Techniques.

[37] Girdhar P., Johri P., Virmani D. (2022). Deep Learning in Image Classification: Its Evolution, Methods, Challenges and Architectures. Advances in Distributed Computing and Machine Learning.

